# Visual Performance in Eyes Undergoing Femtosecond Laser-Assisted Keratoplasty for Advanced Keratoconus

**DOI:** 10.1038/s41598-019-42955-8

**Published:** 2019-04-23

**Authors:** Kazutaka Kamiya, Masahide Takahashi, Akihito Igarashi, Nobuyuki Shoji

**Affiliations:** 1 School of Allied Health Sciences, Kitasato University, Sagamihara, Kanagawa Japan; 2 Department of Ophthalmology, Kitasato University, Sagamihara, Kanagawa Japan; 3 Department of Ophthalmology, Sanno Hospital, Tokyo, Japan

**Keywords:** Corneal diseases, Vision disorders

## Abstract

This study was aimed to compare visual performance in eyes having femtosecond laser-assisted keratoplasty (FLAK) for grade 4 keratoconus and keratoconic eyes according to the Amsler-Krumeich classification. We comprised 15 eyes of 15 patients undergoing FLAK for grade 4 keratoconus and 69 of 69 keratoconic patients (grade 1; 26 eyes, 2; 17 eyes, 3; 10 eyes, and 4; 16 eyes), and compared best spectacle-corrected visual acuity (BSCVA), corneal astigmatism (CA), corneal densitometry (CD), and corneal higher-order aberrations (CHOAs) using the Scheimpflug rotating camera. BSCVA in the post-FLAK group was significantly better than that in grade 3 or 4 group, but not than that in grade 1 or 2 group. CA was significantly lower than that in grade 3 or 4 group, but not than that in grade 1 or 2 group. CD was significantly higher than that in grade 1, 2, and 3 group, and significantly lower than that in grade 4 group. CHOAs were significantly lower than that in grade 3 or 4 group, but not than that in grade 1 or 2 group. FLAK showed significantly better BSCVA, and less CA and CHOAs, than grade 3 or 4 keratoconus, and showed less CD than grade 4 keratoconus.

## Introduction

The femtosecond laser is one of the most revolutionary inventions in medical technology. A recent breakthrough in this technology has enabled us to apply for corneal transplantation. Indeed, femtosecond laser-assisted penetrating keratoplasty (FLAK) has been reported to induce significantly less corneal astigmatism, and to provide faster visual recovery, than manual penetrating keratoplasty, presumably because the geometry of the donor-recipient matching is more physiological and requires less tight sutures^1,2^. Considering that advanced keratoconic eyes requiring FLAK tended to have a large amount of corneal astigmatism, due to the focal anterior protrusion and thinning of the cornea^3,4^, the advantages of FLAK over conventional penetrating keratoplasty for such eyes may be prominent in terms of astigmatic correction. However, no comparative study on the detailed visual performance in such post-FLAK eyes and keratoconic eyes has so far been conducted. It may give us intrinsic insights on the surgical indication of FLAK for advanced keratoconus, especially from the viewpoint of visual quality. The purpose of the present study is to compare the detailed visual performance after FLAK for advanced keratoconus with that of various stages of the disease according to the Amsler-Krumeich classification.

## Results

### Study population

The patient demographics in the post-FLAK and keratoconus grade 1 to 4 groups are summarized in Table 1. In the post-FLAK group, all surgical procedures were uneventful, but postoperatively, mild graft rejection and increased intraocular pressure developed in 1 eye. This eye was followed with appropriate medical therapy, and resolved thereafter. No other vision-threatening complications were seen at any time in post-FLAK eyes.

**Table 1:** Patient demographics of the study population.

	Post-FLAK	Grade 1	Grade 2	Grade 3	Grade 4
Eyes	15	26	17	10	16
Age (years)	35.5 ± 17.3	33.7 ± 11.8	30.4 ± 10.4	28.4 ± 16.0	33.8 ± 11.5
Gender (Male: Female)	12 : 3	15 : 11	10 : 7	7 : 3	9 : 7
LogMAR CDVA	0.11 ± 0.16	0.01 ± 0.15	0.17 ± 0.40	0.30 ± 0.17	0.58 ± 0.63
Corneal astigmatism (D)	3.69 ± 2.26	2.52 ± 1.30	3.94 ± 1.87	6.98 ± 2.50	7.12 ± 3.68
Corneal densitometry (GSU)	23.6 ± 3.0	17.9 ± 3.7	17.3 ± 4.4	18.8 ± 5.2	37.6 ± 19.2
Corneal HOAs (µm)	1.09 ± 0.37	0.78 ± 0.43	1.17 ± 0.59	1.58 ± 0.53	2.03 ± 1.33

FLAK = femtosecond laser assisted penetrating keratoplasty, logMAR = logarithm of the minimum angle of resolution, CDVA = corrected distance visual acuity, D = diopter, GSU = gray scale unit, HOAs = higher-order aberrations.

### Visual acuity

**Figure 1:**
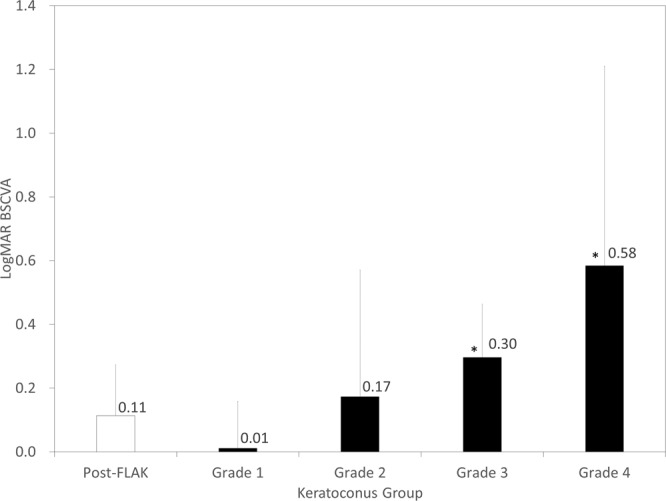
Logarithm of the minimal angle of resolution (logMAR) best spectacle-corrected visual acuity (BSCVA) in post-femtosecond laser-assisted keratoplasty (FLAK) eyes and grade 1 to 4 keratoconic eyes.

The data of logMAR BSCVA in the post-FLAK and the keratoconus grade 1 to 4 groups were shown in Fig. 1. LogMAR BSCVA in the post-FLAK group was significantly better than that in the grade 3 (p = 0.02, Mann-Whitney U test) or the grade 4 group (p = 0.01), but not than that in the grade 1 (p = 0.052) or the grade 2 group (p = 0.51).

### Corneal astigmatism

**Figure 2:**
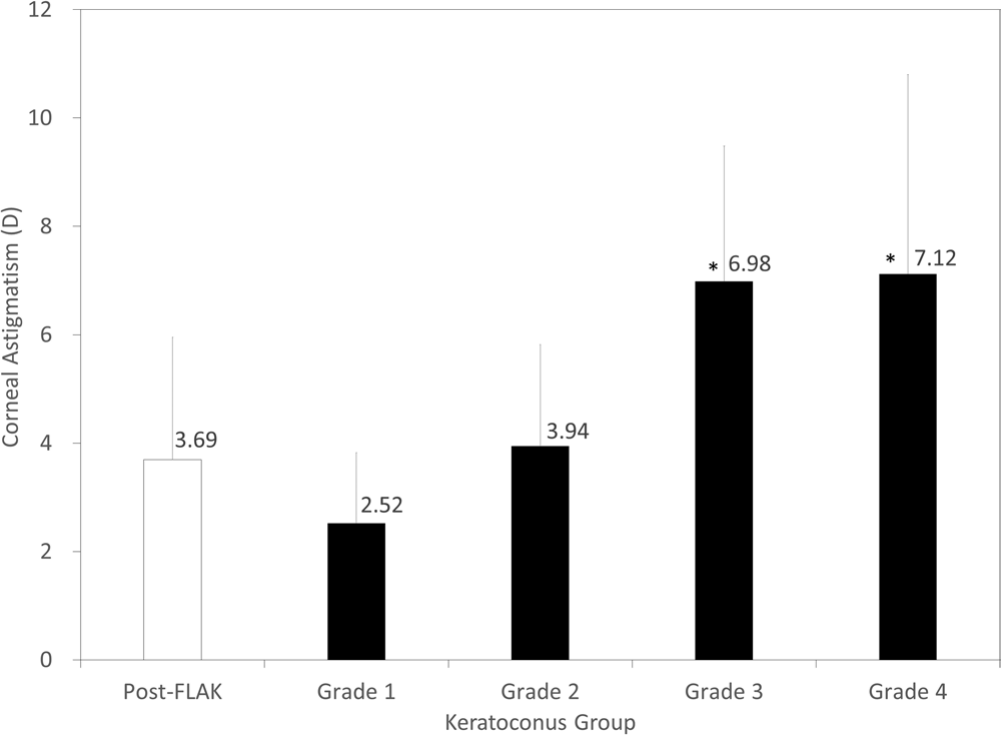
Corneal astigmatism in post-femtosecond laser-assisted keratoplasty (FLAK) eyes and grade 1 to 4 keratoconic eyes. D = diopter.

The data of corneal astigmatism in the post-FLAK and the keratoconus grade 1 to 4 groups were shown in Fig. 2. Corneal astigmatism was significantly lower in the FLAK group than that in the grade 3 (p = 0.007) or the grade 4 group (p = 0.004), but not than that in the grade 1 (p = 0.14) or the grade 2 group (p = 0.42).

### Corneal densitometry

**Figure 3:**
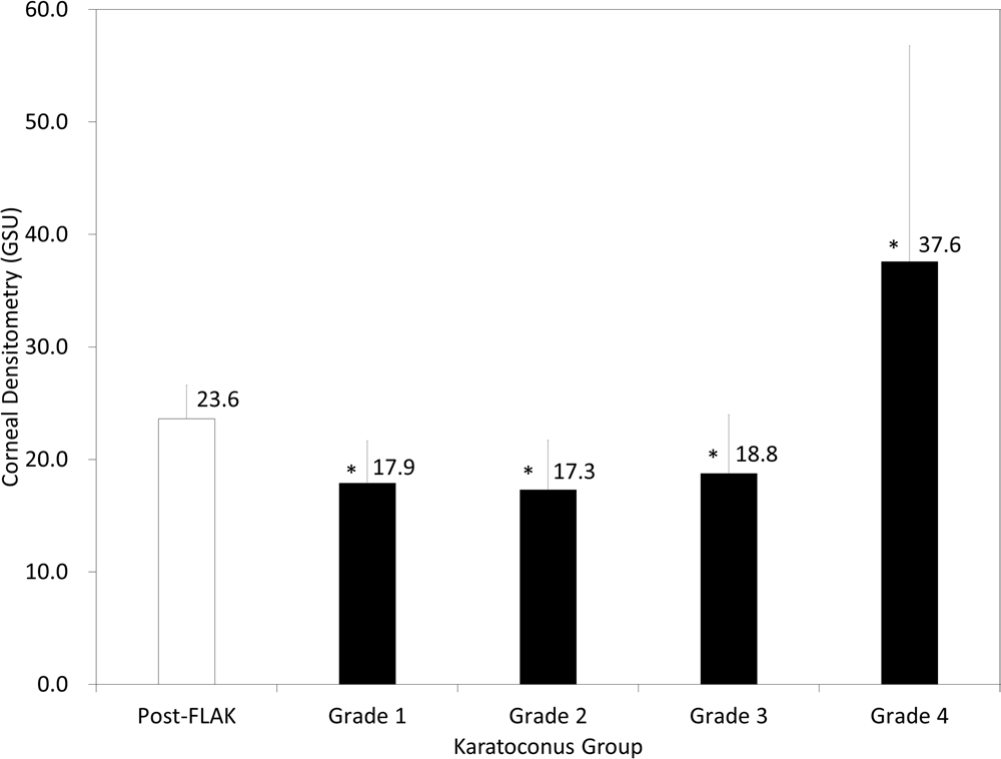
Corneal densitometry in post-femtosecond laser-assisted keratoplasty (FLAK) eyes and grade 1 to 4 keratoconic eyes. GSU = gray scale unit.

The data of corneal densitometry in the post-FLAK and the keratoconus grade 1 to 4 groups were shown in Fig. 3. Corneal densitometry was significantly higher in the FLAK group than that in the grade 1 (p < 0.001), the grade 2 (p < 0.001), and the grade 3 group (p = 0.006), and significantly lower than that in the grade 4 group (p < 0.001).

### Corneal higher-order aberrations

**Figure 4:**
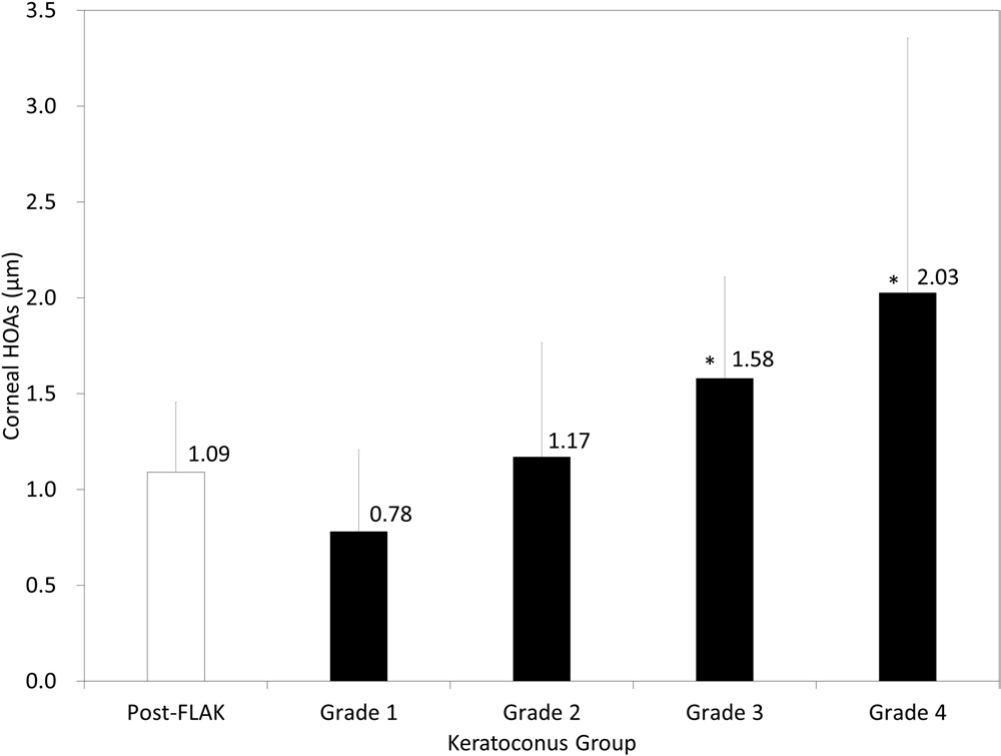
Corneal higher-order aberrations (HOAs) in post-femtosecond laser-assisted keratoplasty (FLAK) eyes and grade 1 to 4 keratoconic eyes.

The data of corneal HOAs in the post-FLAK and the keratoconus grade 1 to 4 groups were shown in Fig. 4. Corneal HOAs were significantly lower in the FLAK group than that in the grade 3 (p = 0.02) or the grade 4 group (p = 0.048), but not than that in the grade 1 (p = 0.06) or the grade 2 group (p = 0.85).

## Discussion

In the present study, our results showed that the post-FLAK keratoconic group provided significantly better BSCVA, and less corneal astigmatism and HOAs, than the keratoconus grade 3 or grade 4 group, and that it provided less corneal densitometry than the keratoconus grade 4 group. To our knowledge, this is the first study to assess the detailed visual performance after FLAK for advanced keratoconus. We believe that this information is clinically helpful for determining the surgical indication of FLAK, especially from the viewpoint of visual quality in keratoconic patients, although we did not consider the possible risk of the intraoperative or postoperative complications, such as graft rejection, intraocular pressure rise, a significant endothelial cell loss, or expulsive hemorrhage.

Until now, there have been several studies on the detailed visual and astigmatic outcomes of FLAK for keratoconus, as listed in Table 2 ^5–8^. Gaster *et al*. demonstrated that BSCVA was 0.44 ± 0.49 logMAR and corneal astigmatism was 4.76 ± 3.41 D 6 months after FLAK^5^. Shivanna *et al*. showed that mean BSCVA was improved to 0.65 and mean corneal astigmatism was decreased by 1.57 D 10 months after FLAK^7^. Chen *et al*. reported that BSCVA was improved, from 0.63 ± 0.19 preoperatively, to 0.19 ± 0.10 logMAR 1 year postoperatively, and that corneal astigmatism was decreased, from 8.12 ± 2.62 D preoperatively, to 3.97 ± 1.06 D 1 year postoperatively^8^. The visual and astigmatic outcomes in the present study were comparable, or slightly better than those in previous studies on FLAK for keratoconus. We assume that the geometry of the donor-recipient matching is more physiological due to highly precise and reproducible incision, and requires less tight sutures, resulting in less corneal astigmatism.

**Table 2:** Previous studies on the visual and astigmatic outcomes of femtosecond laser-assisted penetrating keratoplasty (FLAK) for keratoconus.

Author	Year	Eyes	Age (years)	Follow-up	Laser	Shape	BSCVA (logMAR)	Corneal astigmatism (D)
Gaster *et al*.^8^	2012	54	38.7	6 months	IntraLase	Zigzag	0.44 ± 0.49	4.76 ± 3.41
Yoo *et al*.^9^	2009	1	52	3 weeks	Waveligtht FS200	Mashroom	20/80 (Snellen)	2.91
Shivanna *et al*.^10^	2013	35	N.A.	10 months	Waveligtht FS200	N.A.	0.65 (decimal)	5.81
Chen *et al*.^11^	2015	12	28.0 ± 6.7	1 year	VisuMax	Straight 90°	0.19 ± 0.10	3.97 ± 1.06
Current	2019	15	35.5 ± 17.3	1 year	VisuMax	Straight 90°	0.11 ± 0.16	3.69 ± 2.26

BSCVA = best spectacle-corrected visual acuity, logMAR = logarithm of the minimum angle of resolution, D = diopter, N.A. = not applicable.

With regard to the backward scattering of the cornea, it is rationale that corneal densitometry in eyes having FLAK was significantly lower than that in grade 4 keratoconic eyes, possibly because the grade 4 keratoconus mostly had anterior stromal scar formation. Patel *et al*. mentioned that light scattered from the cornea increases with time after PK and is associated with decreased high- and low-contrast vision^9^. Koh *et al*. stated that corneal density in eyes undergoing PK was significantly higher than in healthy eyes^10^. We recently showed that corneal densitometry and HOAs in post-FLAK patients were significantly larger than those in age-matched healthy subjects^11^. Corneal densitometry in eyes having FLAK was significantly higher than that in grade 1 to 3 keratoconic eyes without corneal scar formation. Our findings were in line with these previous findings demonstrating that penetrating keratoplasty induces some additional corneal scattering, even with and without the use of the femtosecond laser.

There are ongoing concerns about visual quality after FLAK and deep anterior lamellar keratoplasty (DALK) for advanced keratoconus. In the present study, we did not select femtosecond laser-assisted DALK, instead of FLAK, in the surgical population, since most eyes developed acute hydrops due to the rupture of the Descemet’s membrane before FLAK. Chen *et al*. showed that there were no significant differences in BSCVA between the FLAK and femtosecond laser-assisted DALK with baring the Descemet’s membrane groups, and that BSCVA after FLAK was significantly better than that after femtosecond laser-assisted DALK without baring the Descemet’s membrane^8^. They also demonstrated that there were no significant differences in corneal astigmatism between the FLAK and femtosecond laser-assisted DALK groups^8^. Moreover, DALK, especially without baring the Descemet’s membrane, may induce an additional scattering due to the occurrence of interface haze formation. A further study is necessary to assess the detailed visual performance in post-FLAK eyes and post-DALK eyes for advanced keratoconus.

There were at least two limitations to this study. First, this study was performed in a retrospective fashion, and thus the items in the patient backgrounds were not fully matched, especially in terms of the grade of the disease (grade 4 vs. grade 1 to 4). However, we believe that this study provided a good support for determining the surgical indication of FLAK, especially in consideration of visual function in such keratoconic eyes. Second, the sample size was relatively small in this study, since we selected the patients undergoing FLAK, instead of DALK, for grade 4 keratoconus. A prospective randomized controlled study with a large number of keratoconic patients is still necessary to confirm the authenticity of our results.

In conclusion, our comparative study support the view that FLAK for grade 4 keratoconus showed significantly better BSCVA, and less corneal astigmatism and HOAs, than the grade 3 or grade 4 keratoconus, and showed less corneal densitometry than the grade 4 keratoconus. Although we did not take the possible risk of the adverse events of FLAK into consideration, these findings will be clinically helpful for determining the surgical indication of FLAK for advanced keratoconus, especially in terms of visual performance in such patients.

## Methods

### Study population

The study protocol was registered with the University Hospital Medical Information Network Clinical Trial Registry (000031030). Fifteen eyes of 15 patients (12 men and 3 women) who underwent FLAK by one experienced surgeon (KK) for advanced keratoconus due to a rigid gas permeable lens intolerance, and 69 eyes of 69 keratoconic patients (41 men and 28 women), were included in this observational study. Keratoconus was diagnosed with evident findings characteristic of keratoconus (e.g., corneal topography with asymmetric bow-tie pattern with or without skewed axes), and at least one keratoconus sign (e.g., stromal thinning, conical protrusion of the cornea at the apex, Fleischer ring, Vogt striae, or anterior stromal scar) on slit-lamp examination^12^. The keratoconus population was divided into 4 subgroups; grade 1 (26 eyes), grade 2 (17 eyes), grade 3 (10 eyes), and grade 4 (16 eyes) keratoconus groups, according to the Amsler-Krumeich classification, based on astigmatism, corneal power, corneal transparency, and corneal thickness^13^. All pre-FLAK eyes was classified as grade 4 keratoconus due to the presence of anterior stromal scar formation. Eyes with other corneal diseases, and previous ocular trauma or surgery were excluded from the study. Written informed consent was obtained from all patients for the FLAK surgery. This retrospective review of the clinical charts was approved by the Institutional Review Board at Kitasato University and was performed in accordance with the Declaration of Helsinki. Our Institutional Review Board waived the requirement for informed consent for this retrospective study. Patient data was anonymized before access and/or analysis.

### Surgical procedures

For FLAK, we used the VisuMax femtosecond laser system (Carl Zeiss Meditec AG, Jena, Germany) with a 500 kHz repetition rate, as described previously^14^. In brief, the donor cornea was mounted on an artificial anterior chamber, and brought to the femtosecond laser. The femtosecond laser parameters were as follows: donor graft size 6.9 to 7.5 mm, recipient graft size 6.7 to 7.3 mm, 300 nJ power, with side cut angles at 90°, and spot size 3 µm. The donor cornea was oversized by 0.2 mm in all cases. An uncut gap of 25 µm was remained and it was gently dissected by a blunt hook, and then the recipient corneal button was totally excised with curved scissors in the operating room. We placed 16 interrupted sutures, or 8 interrupted 10-0 nylon sutures and a single continuous 16-bite suture. Postoperatively, steroidal (0.1% betamethasone) and antibiotic (1.5% levofloxacin) medications were topically administered 4 times daily for 1 month, and then the frequency was gradually reduced. A suture removal was not routinely done, but loose sutures were removed upon diagnosis in all subjects.

### Assessment of visual acuity, corneal astigmatism, densitometry, and higher-order aberrations

We quantitatively evaluated logarithm of the minimum angle of resolution (logMAR) best spectacle-corrected visual acuity (BSCVA), corneal astigmatism, corneal densitometry, and corneal higher-order aberrations (HOAs) in post-FLAK eyes (at 1 year postoperatively) and in keratoconic eyes. Visual acuity measurement was performed using a Snellen chart with Japanese letters at a distance of 5 m with best correction. Corneal astigmatism, corneal astigmatism, and corneal HOAs were measured with a rotating Scheimpflug imaging instrument (Pentacam HR^TM^, Oculus, Wetzlar, Germany). In brief, the patient was asked to open both eyes and stare at the fixation target on the black background in the center of the blue fixation beam. After attaining alignment, the instrument automatically took 25 Scheimpflug images within 2 seconds. After we checked image quality was checked, we selected only one examination with a high quality factor for each eye. Corneal astigmatism was determined as the difference in simulated keratometry between the flattest and steepest meridians on the central 15° ring (equal to the 3.0-mm ring) around the corneal apex. Corneal densitometry was determined as a measure of the backward scattering of the cornea, and was expressed in grayscale units (GSU). This scale is calibrated by proprietary software, which defines a minimum light scatter of 0 (maximum transparency) and maximum light scatter of 100 (minimum transparency). We used the scale for the whole layers of the cornea within a 2-mm central zone from the corneal apex. Total corneal HOAs were calculated as the root-mean-square of the third- and fourth-order Zernike coefficients for a 4-mm pupil.

### Statistical analysis

All statistical analyses were performed using a commercially available statistical software (Bellcurve for Excel, Social Survey Research Information Co, Ltd., Tokyo, Japan). The normality of all data samples was first checked by the Kolmogorov-Smirnov test. Since the data did not fulfill the criteria for normal distribution, the Mann-Whitney U test was used to compare the data between the two groups. Unless otherwise indicated, the results are expressed as mean ± standard deviation, a value of p < 0.05 was considered statistically significant.
